# Preparation, characterisation and antioxidant activities of rutin-loaded zein-sodium caseinate nanoparticles

**DOI:** 10.1371/journal.pone.0194951

**Published:** 2018-03-26

**Authors:** Shuangling Zhang, Yue Han

**Affiliations:** College of Food Science and Engineering, Qingdao Agricultural University, Qingdao, Shandong Province, China; VIT University, INDIA

## Abstract

Novel rutin-loaded zein-sodium caseinate nanoparticles (ZP) with antioxidant activity in aqueous medium were investigated. The results showed that the sodium caseinate concentrations, dosages of rutin and ethanol volume fractions significantly affected the zein nanoparticles’ characteristics. Concerning the antioxidant properties, the highest values of rutin loaded ZP obtained using 2, 2-diphenyl-1-picrylhydrazyl scavenging and 2 and 2'-azino-bis (3-ethylbenzothiazoline-6-sulphonic acid) decolourisation assays were 52.7% and 71.2%, respectively, and the total antioxidant capacity was 0.40 nmol g^-1^. The results suggest that zein-sodium caseinate nanoparticles can be used as a new nano carrier system for rutin or other water insoluble active ingredients.

## Background

Oxidation occurs in all types of food and can lead to ‘off’ flavours. Antioxidants in food can specifically and/or nonspecifically bind with food components, such as proteins and lipids, and can protect food from oxidation [1].

Due to safety concerns, natural antioxidants are more desirable in food matrices than synthetic antioxidants. Natural extracts such as flavonoids are potential alternatives to synthetic additives [2]. The flavonoid rutin is found in many medicinal herbs, fruits, vegetables and other plants [3]. Rutin is considered beneficial due to its potential protective role for multiple diseases related to oxidative stress; however, rutin has poor water solubility, and this characteristic limits its application in the food industry.

Novel antioxidant carrier systems are required to improve the function of natural antioxidants in food matrices. Nanometer-sized food carrier systems have recently been investigated [4]. Zein is a proline-rich protein obtained from corn, and it has been studied as a potential biomaterial for carrier systems [5, 6]. Because it is insoluble in water, zein is a good candidate for the development of natural biopolymeric colloidal particles, which can be used to encapsulate, protect and control the carrier of active ingredients, drugs and micronutrients [7, 8]. Its potential use for the carrier of epigallocatechin gallate [9], vitamin D_3_ [10], lutein [11] and quercetin [12] has also been investigated.

Zein may not be optimal for food applications because its isoelectric point is around pH 6.2 [5]. This means that zein colloidal particles become unstable and aggregate at pH values close to neutral, and this could occur both in the product and in the physiological pH in the intestine. Another major challenge is the protection against aggregation during drying (lyophilisation) in the preparation of redispersible powders.

Sodium caseinate is Generally Recognized as Safe (GRAS) and classified as a food-grade ingredient by the Food and Drug Administration (FDA). It is soluble 50mg/mL in water and has been used in many applications [13]. Ghorbani et al. reported that sodium caseinate improves the solubility characteristics of gum tragacanth particles when a coacervate method is used [14]. Also, this amphiphilic protein adsorbs to the surface of the hordein nanoparticles, reducing their surface hydrophobicity and increasing electrostatic and steric repulsion [15–16]. However, a combination of these two compounds (zein & sodium caseinate) with nanoparticles and encapsulated rutin has not been investigated thus far.

In the present study, rutin-loaded zein-sodium caseinate nanoparticles were prepared using a simple antisolvent precipitation method [7]. The zein nanoparticles produced had various sodium caseinate concentrations, dosages of rutin and ethanol volume fractions, and the results were used to evaluate the preparation process. Furthermore, the antioxidant properties of the rutin-loaded zein-sodium caseinate nanoparticles in aqueous solutions were studied using 2, 2-diphenyl-1-picrylhydrazyl (DPPH) scavenging [17] and 2, 2'-azino-bis (3-ethylbenzothiazoline-6-sulphonic acid) (ABTS) decolourisation assays [18]. The total antioxidant capacity was evaluated using the phosphomolybdenum method for food applications [19].

## Materials and methods

### Materials

Zein (Molecular Weight:2.5×10^4^–4×10^4^), 2, 2-diphenyl-1-picrylhydrazyl (DPPH), (2, 2'-azino-bis (3-ethylbenzothiazoline-6-sulphonic acid) ABTS, rutin and Trolox were purchased from Sigma-Aldrich (St. Louis, MO). Ethanol, sulfuric acid, potassium phosphate, ammonium molybdate and sodium caseinate (SC) were obtained from Basifu Chemical Corp., Ltd. (Tianjin, China). Water purified by a Milli-Q system (ZMQ-S5V001, Millipore, Billerica, MA) was used for all experiments.

### Methods

#### Preparation of zein-SC nanoparticles (ZP)

Powdered zein (1.0 g) was accurately weighed and dissolved into 20 mL of 80% (volume fraction) aqueous ethanol to form a stock solution. The stock solution was diluted with 50 mL of purified water containing SC at concentrations of 0, 10, 15, 20 and 25 mg mL^−1^ to obtain five zein-SC mass ratios (1:0, 1:0.5, 1:0.75, 1:1 and 1:1.25). The dispersions were prepared under continuous stirring (1000 r min^−1^) with a magnetic stirrer (Ret basic, IKA-Works Inc., Wilmington, NC) at room temperature. The solvent was removed from each dispersion under reduced pressure (Rotavapor N-1100, EYELA, Tokyo, Japan) for 10 min at 45°C. The residues were then subjected to centrifugation at 1,788.8 **×**g for 10 min to separate the small quantity of zein that formed larger aggregates. The final dispersions were stored at 4°C until required for particle size, zeta potential measurements and scanning electron microscopy analyses. To obtain solid powder samples, the dispersions were freeze-dried for 24 h (FD-1000, EYELA, Tokyo, Japan). The powders were stored at −20°C before testing.

#### Preparation of rutin-loaded ZP

Rutin (0.1 g) and powdered zein (1.0 g) were accurately weighed and dissolved into 20 mL of 80% (volume fraction) aqueous ethanol to form a stock solution. The next steps were the same as described in preparation of ZP.

#### The influence of the dosage of rutin on the characteristics of ZP

To study the effect of drug loading on the characteristics of ZP, the experiments were repeated holding all variables constant except for the mass of rutin (0–0.2 g), and the mass ratio of zein to SC was 1:1 with 80% (volume fraction) ethanol in all samples. The procedure described in preparation of rutin-loaded ZP was used for all experiments.

#### Investigation of the effects of ethanol

To study the effect of ethanol on the rutin-loaded ZP, the experiments were repeated holding all variables constant except for the ethanol volume fraction (60, 70, 80 and 90%). The mass ratio of zein to SC was 1:1, and a rutin amount of 0.1g was loaded in all samples. The procedure described in preparation of rutin-loaded ZP was used for all experiments.

### Nanoparticles characterisation

#### Particle size and zeta potential

The zeta potential measurements were conducted using a zeta potential analyser (Zetasizer Nano ZS90, Malvern Instruments Ltd, Malvern, UK) at a proper concentration (0.1–0.5% w/v) of the final dispersion. The hydrodynamic size of various ZP and rutin-loaded ZP were also determined using dynamic light scattering. All determinations were in triplicates.

#### Scanning electron microscopy (SEM)

The morphologies of the nanoparticles were analysed by scanning electron microscopy (SEM) (Tecnai G2 Spirit BioTWIN, FEI, Hillsboro, OR, USA) at 12 kV. The dispersions were diluted using purified water, and one drop of the diluted dispersion was placed on a 200-mesh carbon-coated copper grid. Images were taken at selected magnifications, and representative images are provided.

#### Transmission electron microscopy (TEM)

The morphology of the nanoparticles were also analysed with transmission electron microscopy (HT7700 TEM, Hitachi, Japan) at a voltage of 80 kV. The sample was diluted 40-fold following the same procedure as that used for the particle size and ζpotential measurements. An aliquot (5 μL) of the diluted sample was drop-cast onto a carbon-coated copper grid (400 mesh). The grid was then air-dried at room temperature before loading it into the microscope. Images of the biopolymer particles were recorded in randomly selected fields.

#### Fourier transform infrared spectroscopy

Fourier transform infrared spectroscopy (FTIR) was used to study the structural characteristics of the freeze-dried nanoparticles using a Nicolet Nexus IS10 spectrometer (Thermo Fisher Scientific, USA). The FTIR was equipped with a germanium attenuated total reflectance unit [20]. For analysis, 10 mg of a powdered nanoparticle sample was mixed with 1000 mg of potassium bromide (KBr) powder, and the mixture was pressed into a disk. The spectra were acquired at 400–4000 cm^−1^ in 64 scans with a resolution of 4 cm^−1^. All signals were collected against a background spectrum of KBr powder.

#### Particle yield

The freeze-dried powder nanoparticle samples were weighed to calculate the percentage of solid content. The mass of the solid content (m_sample_) divided by the mass input during the preparation (m_input_) provided the yield for the method (Eq 1).

$$Yield(\%)\frac{m_{sample}{}_{}}{m_{input}}\times 100.$$ (1)

#### Encapsulation efficiency (EE)

The amount of encapsulated rutin was determined indirectly based on the difference between the total amount of rutin and the free amount of rutin [21]. To determine the amount of free rutin, 10 mg of freeze-dried rutin-loaded ZP was mixed with 1 mL of purified water. A vortex was mixed for 30 s and then centrifuged at 11,180 ×g for 5 min. To determine the total amount of rutin, another 10 mg of freeze-dried rutin-loaded ZP was mixed with 1 mL of absolute ethanol. The vortex was mixed for 1 h and then centrifuged at 11,180 ×g for 5 min, and the supernatant was retained. Each extraction was repeated three times, and the supernatants for each type of extraction (e.g. into water or ethanol) were combined. The absorbance of each combined supernatant at 510 nm was determined using ultraviolet-visible spectrophotometry (TU-1810DASPC, Beijing Purkinje General Co., Ltd., Beijing, China). The amounts of total and free rutin were determined using a standard curve, which was constructed using standard solutions of 0–3.2 μg mL^**–1**^ rutin in methanol. The EE was then calculated as follows [22–26]:

$$EE(\%)=\frac{Total\;rutin\;in\;freeze‑dried\;ZP‑free\;rutin\;in\;freeze‑dried\;ZP}{Total\;rutin\;in\;freeze‑dried\;ZP}\times 100$$ (2)

#### Antioxidant properties

The DPPH radical scavenging activity (RSA) caused by rutin-loaded ZP was analyzed using a modification of the method used by Shimada, et al. [17]. Rutin-loaded ZP powder (10 mg) was dissolved into 5 mL of purified water. An aliquot (1 mL) of the obtained dispersions was mixed with 2 mL of freshly prepared DPPH solution (40 mg L^**-1**^ in methanol) and allowed to react for 30 min. Control samples were prepared with deionised water rather than the nanoparticle dispersions. The scavenging activity of DPPH was monitored by measuring the decrease in absorbance at 517 nm. The RSA of the nanoparticle suspension was calculated as follows:

$$RSA(\%)=\left[1-\frac{A_{517}(sample)}{A_{517}(control)}\right]\times 100$$ (3)

Where A_**517**_ (sample) represents the absorbance of the rutin-loaded ZP dispersions, and A_**517**_ (control) represents the absorbance of the DPPH solution without nanoparticles.

The antioxidant activity of the rutin-loaded ZP was also evaluated using an ABTS radical (ABTS^•+^) assay. This was performed using the decolourisation assay described by Re et al. [18]. Rutin-loaded ZP powder (8 mg) was dissolved into 10 mL of purified water to prepare the sample dispersions. The radical was generated by the reaction of 7 mmol L^−1^ ABTS in water with 2.45 mmol L^−1^ potassium persulfate (1:1, final concentration). The mixture was incubated in the dark at room temperature for 16 h to obtain stable absorbance values at 734 nm. A diluted ABTS^•+^ solution (1 mL) was added to 200 μL of the sample dispersions, or 0–3 mmol L^−1^ Trolox in ethanol (blank), and the absorbance at 734 nm was recorded (TU-1810DASPC, Beijing Purkinje General Co., Beijing, China). The measurements were performed at 30°C beginning exactly 1 min after initial mixing and continuing in 1 min intervals up to 180 min after mixing. The experiments showed that the dependence of the antioxidant activity on time was linear for the first 6 min. Accordingly, a 6-min reaction time was selected for all solutions. All experiments were carried out in triplicate. The antioxidant activity (%) was calculated as follows:

$$AA(\%)=\left[(A_{734}blank-A_{734}sample)/A_{734}blank\right]\times 100$$ (4)

Finally, the total antioxidant capacity of the rutin-loaded ZP was evaluated using the method described by Prieto [19]. Rutin-loaded ZP powder (8 mg) was dissolved into 10 mL of purified water. Aliquots (0.1 mL) of the rutin-loaded ZP dispersions were mixed with 0.9 mL of a mixed reagent solution (0.6 mol L^−1^ sulfuric acid, 28 mmol L^−1^ sodium phosphate and 4 mmol L^−1^ ammonium molybdate). The dispersion tubes were sealed and incubated in a boiling water bath for 90 min. After incubation, the dispersions were cooled to room temperature, and the absorbance of each solution was measured at 695 nm against the blank solution. The blank solution was 0.9 mL of a mixed reagent solution and 0.1 mL of the same solvent used for the sample, and it was incubated in the same manner as the other samples.

### Statistical analysis

All the measurements were conducted at least in triplicate, and the data are reported as the mean ± standard deviation. The data were analysed using SPSS 21.0 software (IBM Corp., Armonk, NY). The means were compared using an analysis of variance and the least significant difference. The significance level was set at 0.05.

## Results and discussion

### Characteristics of ZP

**Fig 1:**
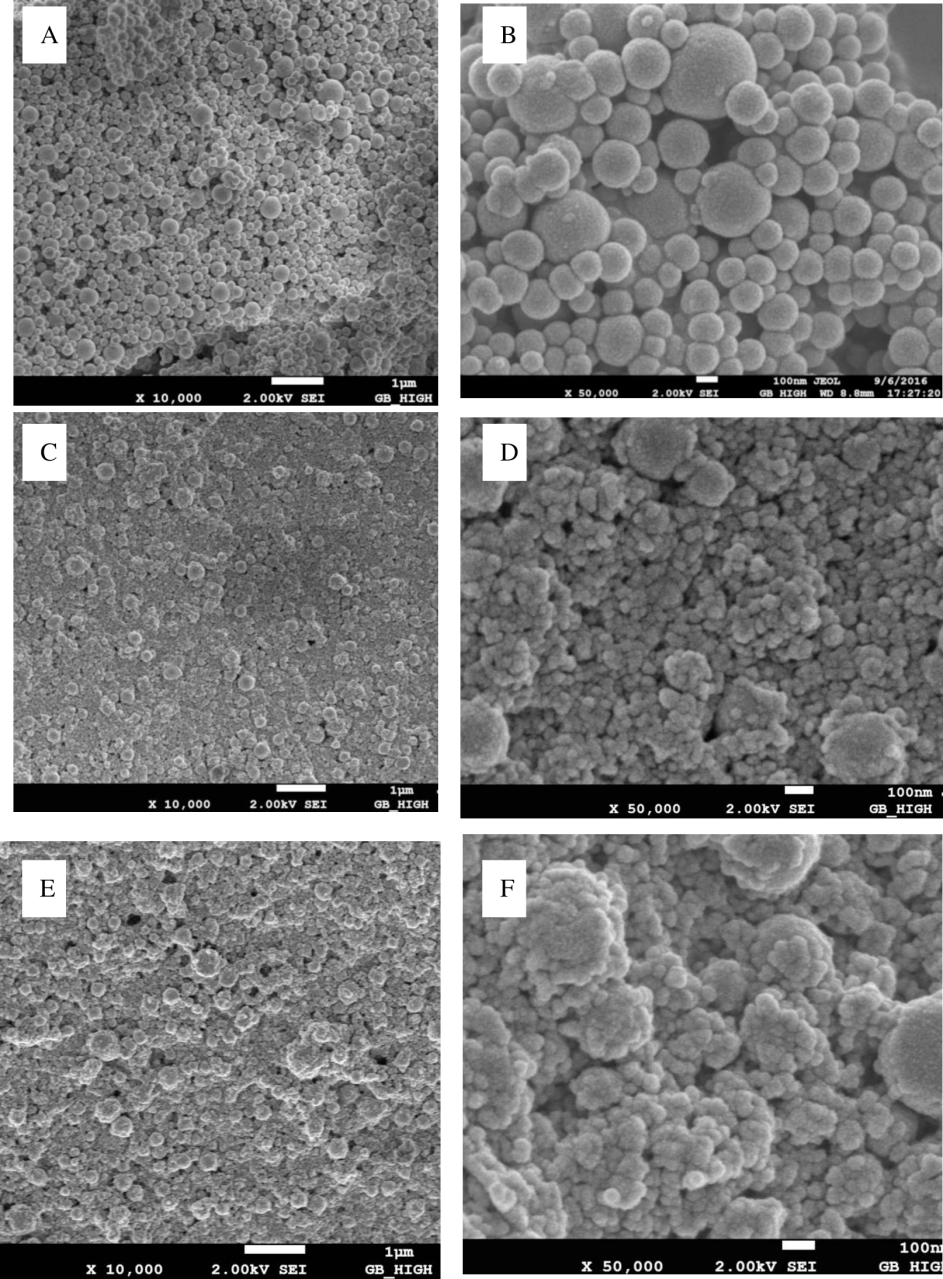
Scanning electron microscopy (SEM) of rutin loaded ZP. Zein (A, B), zein-SC (C, D) and rutin-loaded (0.1g) zein-SC nanoparticles (E, F) at a zein-SC mass ratio of 1: l.

First, the particle size, PDI, zeta potential and particle yield for ZP (Table 1) were investigated, and then scanning electron microscopy images of ZP were obtained (Fig 1A–1D).

**Table 1:** Characterisation of zein-sodium caseinate nanoparticles (ZP) with different zein to SC mass ratios^a^.

Zein to SC mass ratio	Particle size (nm)	Zeta potential (mV)	PDI	Particle yield (%)
1:0	285±1.6^d^	-23.7±0.8^b^	0.17±0.05^b^	51.2±2.1^a^
1:0.5	273±2.3^c^	-25.1±0.2^a^	0.13±0.01^a^^b^	69.4±2.1^b^
1:0.75	265±2.6^b^	-24.9±0.7^a^^b^	0.14±0.03^a^^b^	72.5±1.8^b^
1:1	253±2.3^a^	-23.3±0.9^b^	0.08±0.06^a^	77.3±2.1^c^
1:1.25	249±2.2^a^	-25.7±0.1^a^	0.11±0.03^a^^b^	78.7±1.6^c^

^a^Values are expressed as mean±SD (n = 3); values with different letters in the same column are significantly different (P < 0.05).

In the absence of SC, the zein nanoparticles were uniform spheres and similar sizes (ø 285 nm) with zeta potentials of −23.7 mV. These results were consistent with previous research [12]. After coating with SC, the zeta potential of the zein nanoparticles changed significantly (P < 0.05) to −25.7 mV. This result indicated that the SC was successfully adsorbed onto the zein nanoparticle surface. In Kang-Kang Li’s study [22], zeta potential of NaCas ZP ranged from -34 to -41 mV, our study was coordinate with the previous study. The mass ratio of zein to SC affected the formation and stability of ZP. With a zein to SC mass ratio of 1:0.5, large and small unstable aggregates formed instantaneously after the zein solution was added to the SC aqueous solution, therefore, lower particle yield (69.4%) was obtained. Subsequently, with a zein to SC mass ratio of 1:0.75, small aggregates were observed at the bottom of the vessel after it was kept at an ambient temperature for 5 minutes and a relative higher particle yield (72.5%) obtained. This aggregation might have occurred because the surfaces of the zein nanoparticles were not saturated with SC [27]. For zein to SC mass ratios between 1:1 and 1:1.25, stable dispersions were formed, and the particle yield was between 77.3 and 78.7%. Increasing the zein to SC mass ratio from 1:0.5 to 1:1 resulted in a decrease in the particle size from 273 nm to 253 nm; however, further increases in the mass ratio of zein to SC to 1:1.25 did not result in statistically significant changes in either the particle size or particle yield. These results suggested that at a certain point, the zein nanoparticles become saturated with SC molecules [27] and this was called critical micelle concentration. In this case, critical micelle concentration of SC was the mass ratio of zein to SC 1:1. When zein to SC mass ratios lower 1:1, unstable dispersions were formed and lower particle yield of obtained nanoparticles, while higher 1:1, stable dispersions existed and higher particle yield of obtained nanoparticles. All samples had a PDI of less than 0.2, indicating a narrow size distribution of the nanoparticles.

**Fig 2:**
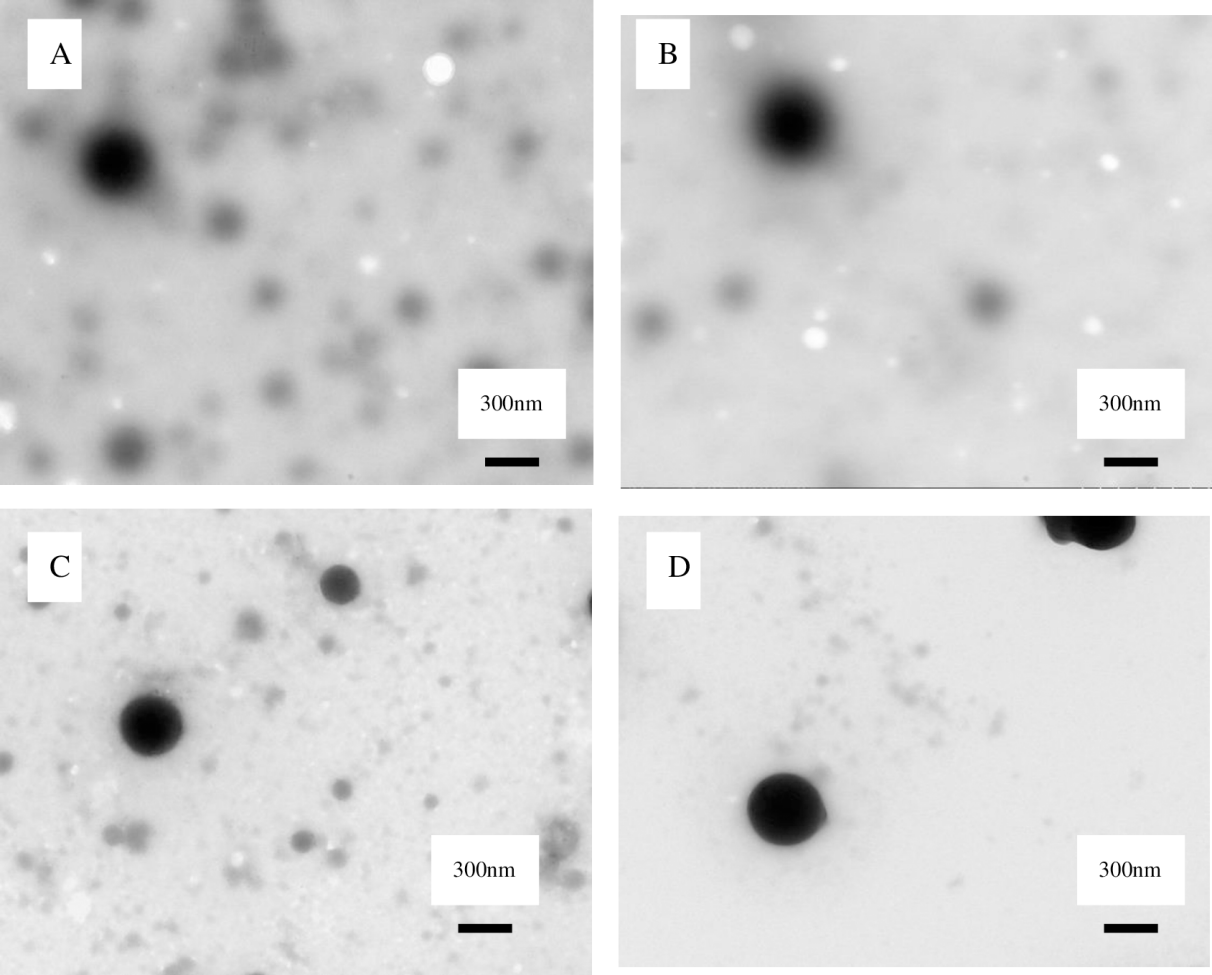
Transmission electron microscopy (TEM) of rutin loaded ZP. A: 0.05 g rutin; B: 0.10 g rutin; C: 0.15 g rutin and D: 0.20 g rutin at a zein-SC mass ratio of 1: l.

Next, the rutin-loaded ZP with 0.1g of rutin was evaluated (Table 2, Fig 1E and 1F, Fig 2).

**Table 2:** Characterisation of rutin-loaded zein-sodium caseinate nanoparticles with different zein to SC mass ratios^a^ (0.1g rutin loaded).

Zein to SC mass ratio	Particle size (nm)	Zeta potential (mV)	PDI	Particle yield(%)	EE (%)
1:0	267±2.4^d^	-23.9±0.8^b^	0.09±0.03^a^	51.4±1.7^a^	57.3±1.6^a^
1:0.5	261±2.2^c^	-25.5±0.9^a^	0.09±0.01^a^	74.8±1.6^a^^b^	59.4±1.2^a^b
1:0.75	256±1.5^b^	-23.7±0.7^b^	0.16±0.02^b^	80.5±1.4^b^	61.1±1.8^b^
1:1	248±2.3^a^	-25.6±0.5^a^	0.11±0.03^a^^b^	84.2±1.5^b^	68.1±1.2^c^
1:1.25	247±2.8^a^	-23.9±0.5^b^	0.14±0.01^a^^b^	87.6±1.4^a^^b^	69.5±1.6^c^

^a^Values are expressed as mean±SD (n = 3); values with different letters in the same column are significantly different (P < 0.05).

When rutin was loaded, the nanoparticles formed both small and large spheres (Fig 1E and 1F, Fig 2A–2D). The particle size, PDI, zeta potential and particle yield all showed similar trends to ZP. As the mass ratio of zein to SC decreased, the particle size decreased from 261 nm to 247 nm, the PDI remained stable at between 0.09 and 0.16 and the zeta potential changed from −23.7 to −25.6 mV. The mass ratio of zein to SC affected the EE of the rutin-loaded ZP, with an increase in the zein to SC mass ratio from 1:0.5 to 1:1.25, which resulted in an increase in the EE from 59.4 to 69.5%. The EE remained stable when the zein to SC mass ratio was increased from 1:1 to 1:1.25. These results suggest that adding more SC would not be beneficial for the encapsulating rutin of ZP [27]; this was also because of critical micelle concentration of SC.

### Effect of ethanol volume fraction in the stock dispersions

The particle size, zeta potential, particle yield and EE were investigated for rutin-loaded ZP produced with stock dispersions containing 0.1g of rutin and 1g of zein dissolved into 20 mL of 60–90% ethanol (Table 3).

**Table 3:** Effect of different concentrations of ethanol on the characteristics of rutin-loaded zein-sodium caseinate nanoparticles^a^ (zein to SC mass ratio 1:1, 0.1g rutin loaded).

Concentrations of ethanol (%)	Particle size (nm)	Zeta potential (mV)	PDI	Particle yield (%)	EE (%)
60	265±2.06 ^c^	-24.7±0.72 ^b^	0.11±0.04 ^a^	76.7±1.57 ^a^	60.8±1.38 ^a^
70	258±1.69 ^b^	-24.4±0.62 ^b^	0.12±0.03 ^a^	79.1±1.85 ^a^	65.3±1.54 ^b^
80	248±2.27 ^a^	-25.6±0.46 ^b^	0.11±0.03 ^a^	84.2±1.54 ^b^	68.1±1.19 ^c^
90	245±1.64 ^a^	-28.9±1.04 ^a^	0.17±0.02 ^a^	86.6±1.47 ^b^	71.6±0.75 ^d^

^a^Values are expressed as mean±SD (n = 3); values with different letters in the same column are significantly different (P < 0.05).

The particles produced at higher ethanol volume fractions were smaller than those produced with lower ethanol volume fractions. For example, when the ethanol volume fraction was increased from 60 to 80%, the particle size decreased significantly (P < 0.05) from 264 to 248 nm. These results are consistent with earlier research on nanoparticles synthesised from soy protein [10]. Physically, these observations may be explained by competing particle formation mechanisms. When stock dispersions with lower ethanol volume fractions (e.g. <60% ethanol) are homogenised, the ethanol concentration in the dispersed droplets decreases to the solubility limit for zein and rutin faster than for stock dispersions with higher ethanol volume fractions. In this case, the zein in the dispersed droplets solidifies quickly before the droplets can be sheared into smaller sizes. This could result in the formation of bigger particles from stock dispersions with lower ethanol volume fractions than from those with higher ethanol volume fractions. As the volume fraction of ethanol was increased from 70 to 80%, the particle yield increased significantly (P < 0.05) from 79.1 to 84.3%; however, further increases in the ethanol volume fraction to 90% resulted in a little increase in the particle yield (to 86.6%) that was not significant (P < 0.05). When the ethanol volume fraction was increased from 60 to 90%, the EE increased significantly (P < 0.05) from 60.8 to 71.6% (Table 3). These results suggest that rutin and zein are more soluble in solutions with higher ethanol volume fractions. All samples had relatively stable zeta potentials (−28.9 to −24.7 mV), indicating that the SC coating was stable. In addition, all samples had a PDI of less than 0.2, indicating a narrow size distribution for the nanoparticles.

### Effect of dosage of rutin in the stock dispersions

Rutin-loaded ZP with different dosages of rutin was evaluated (Table 4). All samples were small (246–255 nm), with PDIs of 0.09–0.14 and zeta potentials between −28.0 and −23.3 mV.

**Table 4:** The influence of the dosages of rutin on the characteristics of zein-sodium caseinate nanoparticles^a^ (zein to SC mass ratio 1:1, 80% concentrations of ethanol).

Dosage of rutin (g)	Particle size (nm)	Zeta potential (mV)	PDI	Particle yield (%)	EE (%)	LA (%)
0	253±2.3^b^	-23.3±0.9^d^	0.08±0.06^a^	77.3±2.1^a^	—-	—-
0.05	246±2.0^a^	-23.5±0.7^d^	0.11±0.40^a^	79.6±1.6^a^^b^	48.4±1.3^a^	1.2
0.1	248±2.2^a^^b^	-25.6±0.5^c^	0.11±0.03^a^	84.2±2.4^c^	68.1±1.2^b^	1.5
0.15	250±2.9^b^	-26.9±0.5^b^	0.09±0.01^a^	84.4±1.5^c^	70.0±1.3^bc^	1.6
0.2	255±2.1^b^	-28.0±0.6^a^	0.14±0.04^b^	82.2±2.2^bc^	70.6±1.2^c^	2.8

^a^Values are expressed as mean±SD (n = 3); values with different letters in the same column are significantly different (P < 0.05).

Compared with the nanoparticles with no rutin (Table 1), the particle size decreased significantly (P < 0.05). This could have occurred because the rutin affected the crystalline state of the aggregated particles [28]. Rutin acts as a plasticizer between zein-SC nanoparticles, and when a lower quantity of rutin was added, the zein nanoparticles were more uniform and stable and were smaller. These results are consistent with the literature [29]. When the dosage of rutin increased from 0.10 to 0.20g, the particle size increased no significantly (P < 0.05) from 248 to 255 nm, which could be attributed to the higher content of encapsulated rutin.

When the dosage of rutin was increased from 0.05 to 0.2 g, the EE increased from 48.4 to 70.6% (Table 4). By comparison, the EEs for 5-fluorouracil in zein nanoparticles were 6.9–68% [30], and the gitoxin in zein nanoparticles was 1.8–21% [31]. The EE for vitamin D_3_ in zein nanoparticles was 52–88% with a vitamin D_3_ to zein mass ratio of 0.075:1 [20], and that for thymol in zein nanoparticles was 7.02% [32].

In order to quantify loading efficiency (LA), the actual weight of rutin per gram of the rutin loaded ZP was calculated by a TGA heat testing (TG209F3, NETZSCH, Germany).The LA was calculated by dividing the amount of rutin weight loss during heating by the rutin loaded ZP amount [4].

For the particle yield, an increase from 79.6% to 84.4% was observed as the dosage of rutin was increased from 0.05 to 0.15. This suggests that the rutin inhibited the aggregation and precipitation of nanoparticles. For LA, the loading efficiency of rutin was 1.2%, 1.5%, 1.6%, 2.8% respectively.

### Antioxidant activity in aqueous medium

The antioxidant activities of the rutin-loaded ZP in aqueous medium were evaluated using DPPH scavenging and ABTS decolourisation assays, and the total antioxidant capacity was evaluated using the phosphomolybdenum method (Table 5).

**Table 5:** Antioxidant characteristics of rutin-loaded zein-SC nanoparticles with different dosages of rutin (zein to SC mass ratio 1:1, 80% concentrations of ethanol)^a^.

Dosage of rutin (g)	DPPH (%)	ABTS (%)	Phosphomolybdenum method (nmol/g)
0	22.3±1.8^a^	24.3±7.9^a^	0.26±0.08^a^
0.05	43.3±0.6^b^	52.5±2.9^b^	0.35±0.01^b^
0.1	48.8±1.7^c^	53.5±0.6^b^	0.39±0.01^c^
0.15	51.6±2.9^cd^	69.6±0.4^c^	0.39±0.01^c^
0.2	52.7±0.3^d^	71.2±1.6^c^	0.40±0.01^c^

^a^Values are expressed as mean±SD (n = 3); values with different letters in the same column are significantly different (P < 0.05).

Due to the antioxidant activity of zein, even ZP showed some activity in the DPPH assay (22.3%), ABTS assay (24.3%) and the total antioxidant capacity test (0.26 nmol g^-1^). When rutin was loaded to the nanoparticles, the antioxidant activity in the aqueous solutions increased significantly (P < 0.05). With a dosage of rutin of 0.20g, the DPPH assay, ABTS assay and total antioxidant capacity test results were 52.7%, 71.2% and 0.40 nmol g^-1^, respectively. The DPPH assay, ABTS assay and the total antioxidant capacity test of rutin loaded ZP (0.20g rutin) was approximately 2.5, 3 and 1.5 times of ZP. These results showed that rutin encapsulated in ZP could still act as a strong antioxidant, and this activity could occur due to rutin on the surface or near the edges of the nanoparticles.

Zein alone was able to inhibit the free radicals in a related study by Zhang et al. (2011) [33]. The results indicated that zein had slight antioxidant capacity (less than 25% DPPH reduced after 24 h). The zein was also measured in this study at the same concentration and 22.3% DPPH inhibited was obtained after 30 min. Wu et al. (2012) report that 0.67 mg ml^-1^ zein encapsulated carvacrol at pH 4 and 10 inhibited 50% of DPPH [25]. In our study, 52.7% DPPH reduced of rutin loaded ZP (0.20g rutin) was obtained at the same concentration. Our results are in accordance with these results. Besides, rutin loaded ZP prepared was water soluble, which could be used in water medium elsewhere.

### FTIR spectra of the nanoparticles

**Fig 3:**
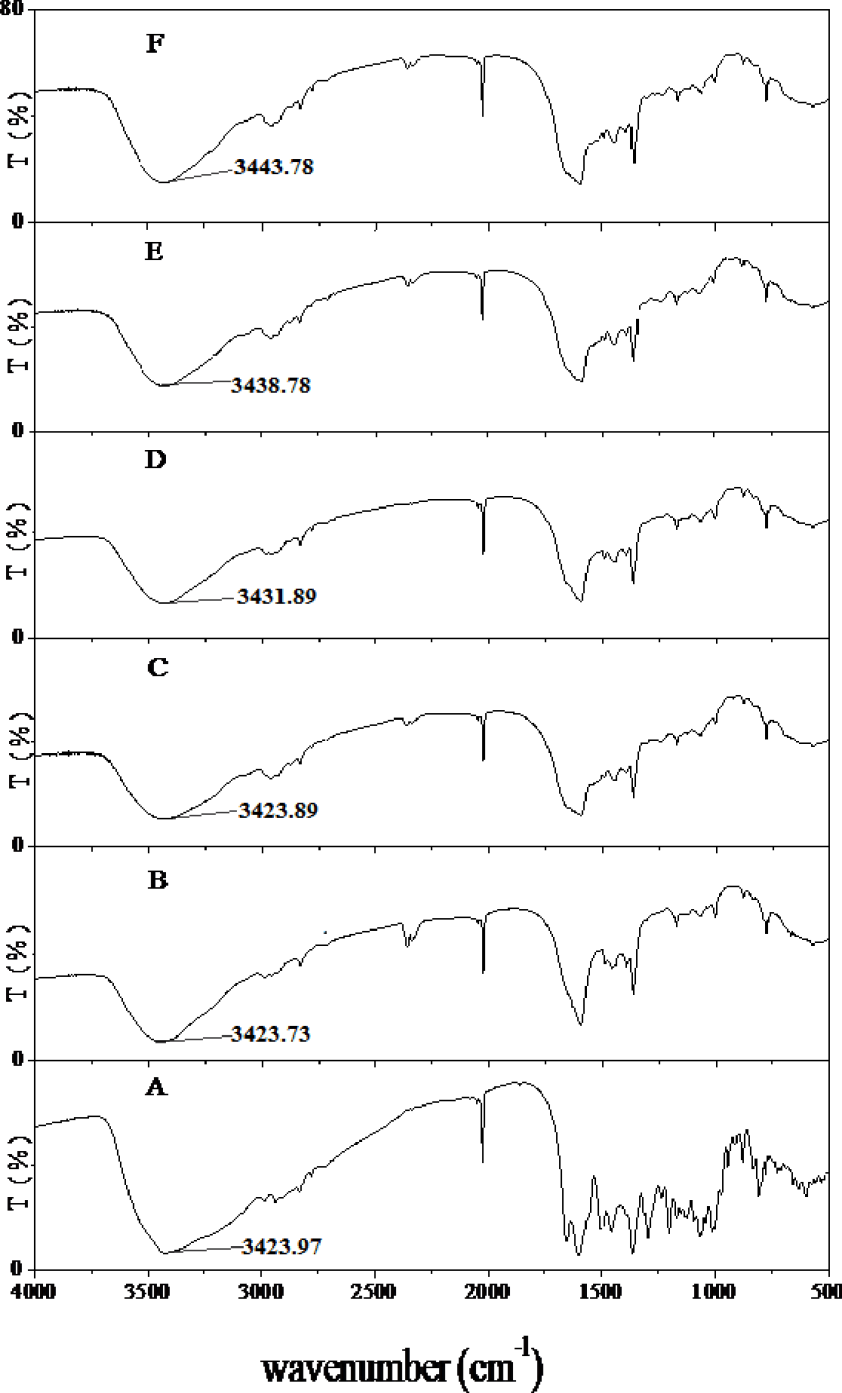
FTIR spectra of powdered zein-SC nanoparticles. A: rutin, B: zein-SC nanoparticles and rutin-loaded zein-SC nanoparticles (C: 0.05g rutin, D: 0.10g rutin, E: 0.15g rutin and F: 0.20g rutin).

FTIR spectra were recorded for powdered rutin, ZP and rutin-loaded ZP with different dosages of rutin (0.05, 0.10, 0.15 and 0.20 g) (Fig 3A–3F).

FTIR spectroscopy can efficiently identify molecules and their surface groups within polymer matrices [20]. The FTIR spectra of the rutin-loaded ZP were similar to those of rutin and ZP, indicating that no chemical reactions occurred between rutin and zein under the studied conditions. The characteristic O‒H stretching vibration of rutin can be observed as a small shoulder at 3423.97 cm^‒1^ in Fig 3A [34]. In the FTIR spectra of the rutin-loaded ZP, the O‒H stretching vibration was observed at approximately 3430 cm^‒1^ (Fig 3C–3F). FTIR spectra can provide information about hydrogen bonding among components in rutin-loaded ZP. The O‒H⋯O stretching peak of the rutin-loaded ZP shifted from 3423 to 3430 cm^‒1^ after rutin was loaded. This suggests an increased hydrogen bonding activity between rutin and ZP. With an increase in the dosage of rutin to 0.20g, the O‒H⋯O stretching peak of the rutin-loaded ZP shifted to 3443.78 (Fig 3F), which indicated a stronger hydrogen bonding activity among components of rutin-loaded ZP [35]. These interactions resulted in a good compatibility between the components of rutin-loaded ZP.

## Conclusions

Novel rutin-loaded zein-SC nanoparticles with antioxidant activity in aqueous medium were prepared. The mass ratios of zein to SC, the volume fractions of ethanol and the dosages of rutin significantly affected the zein nanoparticle characteristics. The rutin-loaded ZP was round with high particle yields (up to 86.6%) and EE (up to 71.6%). The best results obtained from the DPPH and ABTS assays were 52.7% and 71.2%, respectively, and the total antioxidant capacity was 0.40nmol g^-1^. These results suggest that zein-sodium caseinate nanoparticles can be used as a new nano carrier system for rutin or other water insoluble active ingredients.
